# Supplementary material for: Revisiting atypical language lateralization in dyslexia
Source: Brain Commun. 2025 Nov 11;7(6):fcaf444. doi: 10.1093/braincomms/fcaf444 (PMC12680132; doi:10.1093/braincomms/fcaf444)
Supplement: fcaf444_Supplementary_Data [file fcaf444_supplementary_data.pdf]

Supplement to manuscript "*Revisiting atypical language lateralization in dyslexia*".

A. Group-level and between-group fMRI activations during the reading task

Contrast	Anatomical definition	MNI coordinates (peak voxel)				
		X	*Y*	*Z*	*t-value*	*Cluster size*
Control group	**Left precentral gyrus**	**-48**	**10**	**30**	**9.46**	**2966**
	Left inferior frontal gyrus, opercular part	-48	16	10	9.34	
	Left inferior frontal gyrus, triangular part	-28	30	0	8.81	
	**Left inferior occipital gyrus**	**-26**	**-100**	**-10**	**9.46**	**1457**
	Left fusiform gyrus	-36	-47	-18	8.7	
	Left inferior temporal gyrus	-48	-57	-13	8.37	
	**Right lobule VIIB of cerebellar hemisphere**	**19**	**-77**	**-48**	**9.07**	**1020**
	Right lobule VIII of cerebellar hemisphere	26	-72	-50	7.77	
	Right lobule VI of cerebellar hemisphere	9	-77	-20	6.8	
	**Left superior frontal gyrus, dorsolateral**	**-11**	**13**	**54**	**8.63**	**1088**
	Right superior frontal gyrus, medial	9	20	42	8.2	
	Left supplementary motor area	-4	10	57	8.06	
	**Right insula**	**32**	**23**	**-3**	**8.28**	**1019**
	Right inferior frontal gyrus, pars orbitalis	42	26	-10	7.33	
	Right inferior frontal gyrus, triangular part	46	16	22	5.87	
	**Right inferior frontal gyrus, triangular part**	**24**	**-94**	**-6**	**7.89**	**153**
	Right inferior occipital gyrus	42	-82	-10	3.89	
	**Left middle temporal gyrus**	**-54**	**-40**	**7**	**7.71**	**240**
	Left middle temporal gyrus	-44	-42	4	4.63	
	Left middle temporal gyrus	-56	-60	7	4.28	
	**Left anterior cingulate cortex, supracallosal**	**-6**	**3**	**30**	**6.92**	**58**
	**Left middle occipital gyrus**	**-28**	**-70**	**40**	**6.66**	**77**
	**Right calcarine fissure and surrounding cortex**	**16**	**-64**	**12**	**5.63**	**510**
	Left calcarine fissure and surrounding cortex	-16	-77	14	5.49	
	Right calcarine fissure and surrounding cortex	19	-67	4	5.05	
	**Left caudate nucleus**	**-18**	**-12**	**20**	**5.37**	**86**
	Left mediodorsal lateral parvocellular	-8	-12	7	4.96	
Dyslexia group	**Left inferior frontal gyrus, triangular part**	**-34**	**26**	**-3**	**10.33**	**3292**
	Left inferior frontal gyrus, opercular part	-41	8	27	9.3	
	Left temporal pole	-48	13	-13	8	
	**Right insula**	**39**	**26**	**-3**	**9.77**	**742**
	Right insula	32	26	2	9.62	
	Right inferior frontal gyrus, pars orbitalis	36	36	-6	6.09	
	**Left inferior occipital gyrus**	**-24**	**-97**	**-8**	**9.65**	**2249**
	Right calcarine fissure and surrounding cortex	19	-97	-6	9.61	
	Left inferior occipital gyrus	-41	-77	-16	8.55	
	**Left supplementary motor area**	**-1**	**18**	**50**	**9.38**	**927**
	Right middle cingulate & paracingulate gyri	12	28	32	6.93	
	Left supplementary motor area	-1	0	62	6.58	
	**Right lobule VI of cerebellar hemisphere**	**29**	**-67**	**-23**	**8.24**	**1338**
	Right lobule VIII of cerebellar hemisphere	29	-67	-56	8.07	
	Right lobule VIII of cerebellar hemisphere	34	-62	-50	8.01	
	**Left middle temporal gyrus**	**-51**	**-40**	**7**	**5.59**	**123**
	Left middle temporal gyrus	-64	-32	4		
CON>DYS	**Left supramarginal gyrus**	**-66**	**-27**	**37**	**4.43**	**88**
	Left supramarginal gyrus	-64	-34	44	4.23	
	Left supramarginal gyrus	-66	-30	27	4.08	
DYS>CON	**Lobule VI of right cerebellar hemisphere**	**26**	**-62**	**-23**	**4.77**	**64**

Supplementary Table 1. Whole-brain analysis results for the contrast reading > control in the control group (CON, n = 35) and the dyslexia group (DYS, n = 35), as well as between-group comparisons (CON > DYS and DYS > CON). Significant activations were identified at a voxel-level threshold of $p < .001$ (uncorrected) with cluster-level FWE correction at $p < .05$.

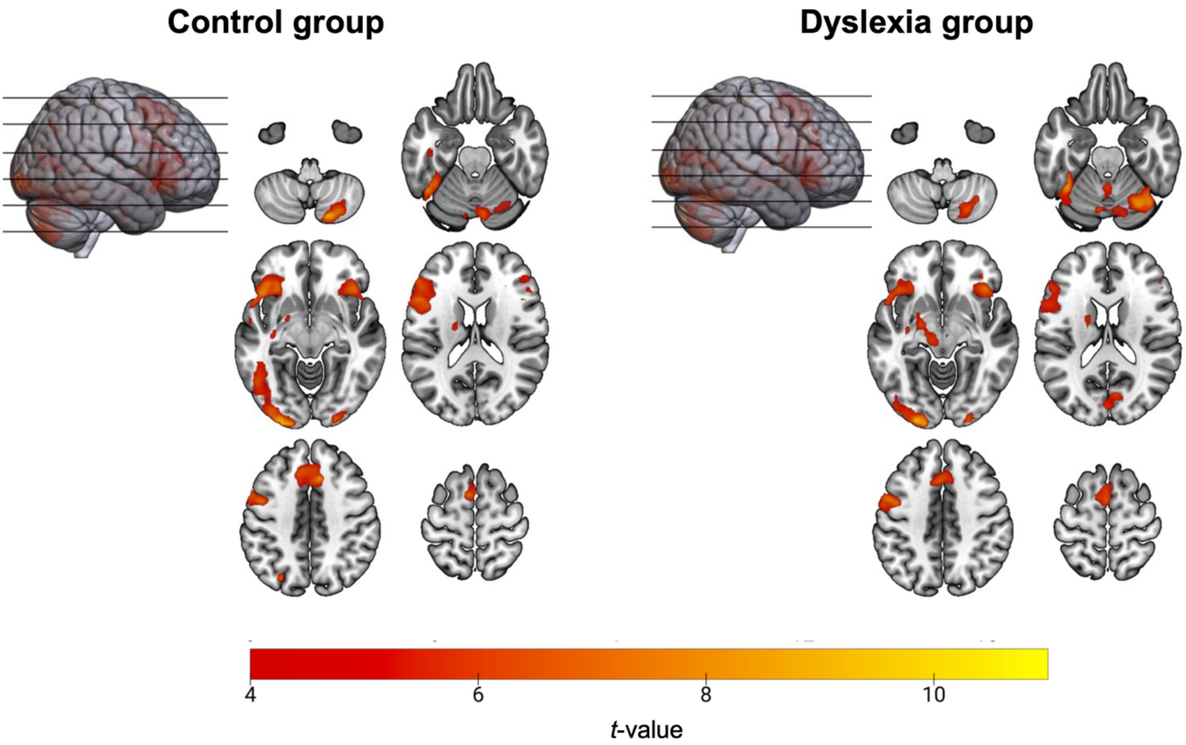

Supplementary Figure 1. Significant fMRI activations based on t-statistic maps during the reading task shown separately for the control group (n = 35) and the dyslexia group (n = 35), voxel-level threshold: p < 0.001 uncorrected; cluster-level threshold: p < 0.05 FWE-corrected. Colour scale bar indicates the magnitude of t-values.

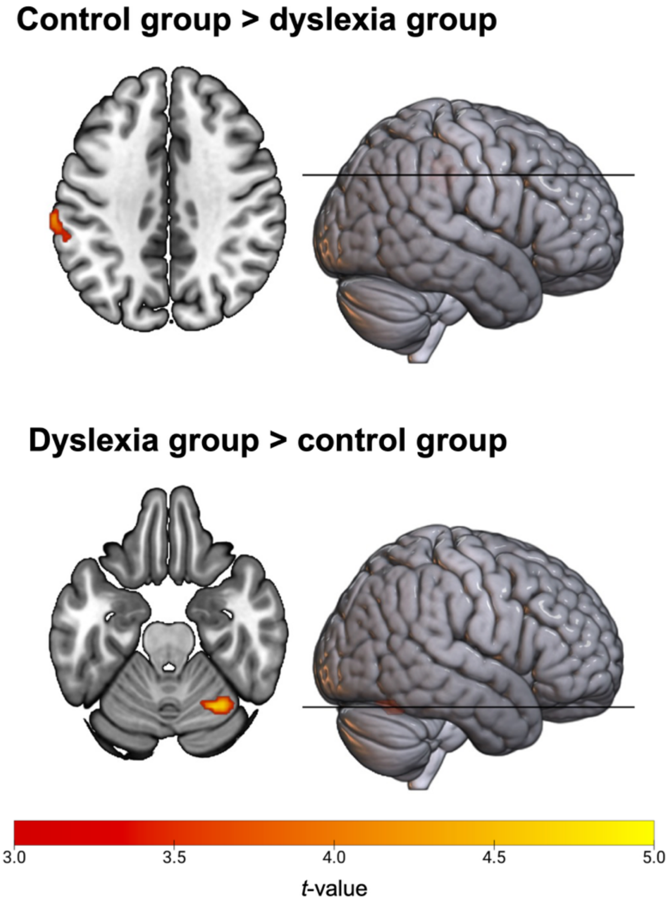

Supplementary Figure 2. Significant between-group fMRI activations based on t-statistic maps during the reading task (controls (n = 35) > dyslexia (n = 35) and dyslexia > controls), voxel-level threshold: p < 0.001 uncorrected, cluster-level threshold: p < 0.05 FWE-corrected. Colour scale bar indicates the magnitude of t-values.

B. Group-level and between-group fMRI activations during the rhyming task

		MNI coordinates (peak voxel)				
**Contrast**	**Anatomical definition**	***X***	***Y***	***Z***	***t-value***	***Cluster size***
**Controls**	**Left inferior temporal gyrus**	**-48**	**-64**	**-8**	**15.78**	**24308**
	Right lobule VIII of cerebellar hemisphere	32	-70	-48	15.15	
	Right lobule VI of cerebellar hemisphere	9	-74	-20	15.03	
	**Left supplementary motor area**	**-6**	**8**	**60**	**14.58**	**1683**
	Right middle cingulate & paracingulate gyri	12	33	30	11.11	
	Left superior frontal gyrus, dorsolateral	-11	13	50	10.96	
	**Left superior temporal gyrus**	**-51**	**-44**	**12**	**7.77**	**242**
	Left middle temporal gyrus	-56	-30	2	5.53	
	Left supramarginal gyrus	-56	-44	24	4.19	
	**Left calcarine fissure and surrounding cortex**	**-16**	**-80**	**10**	**4.54**	**87**
	Left calcarine fissure and surrounding cortex	-11	-70	10	4.28	
**DYS**	**Left inferior frontal gyrus, triangular part**	**-28**	**30**	**2**	**14.65**	**16483**
	Right Insula	34	26	-6	13.64	
	Left red nucleus	-6	-27	-13	13.17	
	**Left supplementary motor area**	**-1**	**20**	**47**	**11.29**	**993**
	Left supplementary motor area	-4	10	52	10.42	
	Right superior frontal gyrus, medial	6	20	42	10.36	
	**Right lobule X of cerebellar hemisphere**	**22**	**-40**	**-43**	**9.39**	**64**
	**Left lobule VIII of cerebellar hemisphere**	**-31**	**-67**	**-53**	**5.58**	**83**
	Left lobule VIIB of cerebellar hemisphere	-38	-62	-53	5.00	
	**Left middle temporal gyrus**	**-56**	**-30**	**2**	**5.39**	**58**
	Left middle temporal gyrus	-54	-40	4	4.26	
**CON>DYS**	**Left precentral gyrus**	**-36**	**8**	**37**	**5.28**	**74**
	Left middle frontal gyrus	-34	16	40	3.83	
	**Left superior frontal gyrus, medial**	**-8**	**30**	**50**	**4.63**	**86**
	Left superior frontal gyrus, dorsolateral	-14	28	34	3.77	
	**Left middle occipital gyrus**	**-28**	**-74**	**22**	**4.18**	**59**
	Left middle occipital gyrus	-26	-72	30	3.85	
**DYS>CON**		-	-	-	-	-

Supplementary Table 2. Whole-brain analysis results for the contrast rhyming > control in the control group (CON, n = 35) and the dyslexia group (DYS, n = 35), as well as between-group comparisons (CON > DYS and DYS > CON). Significant activations were identified at a voxel-level threshold of $p < .001$ (uncorrected) with cluster-level FWE correction at $p < .05$.

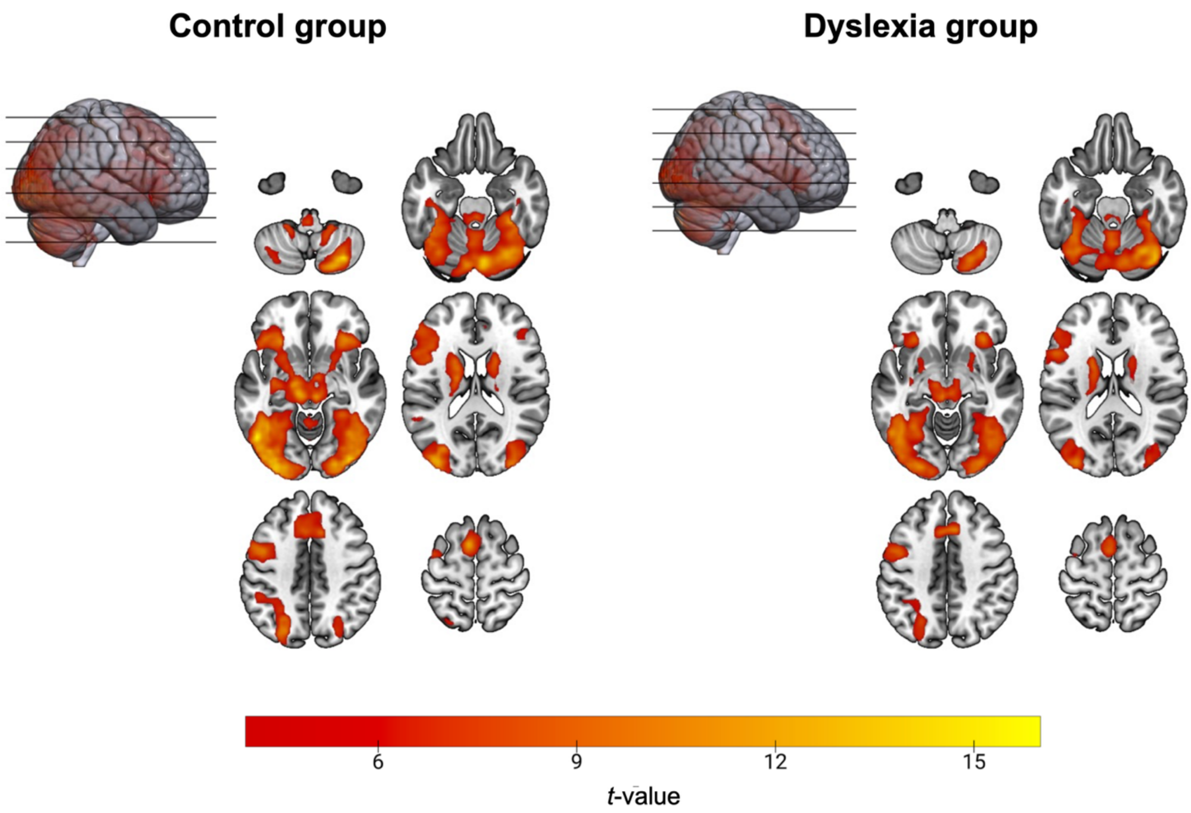

Supplementary Figure 3. Significant fMRI activations based on t-statistic maps during the rhyming task shown separately for the control group (n = 35) and the dyslexia group (n = 35), voxel-level threshold: $p < 0.001$ uncorrected; cluster-level threshold: $p < 0.05$ FWE-corrected. Colour scale bar indicates the magnitude of t-values.

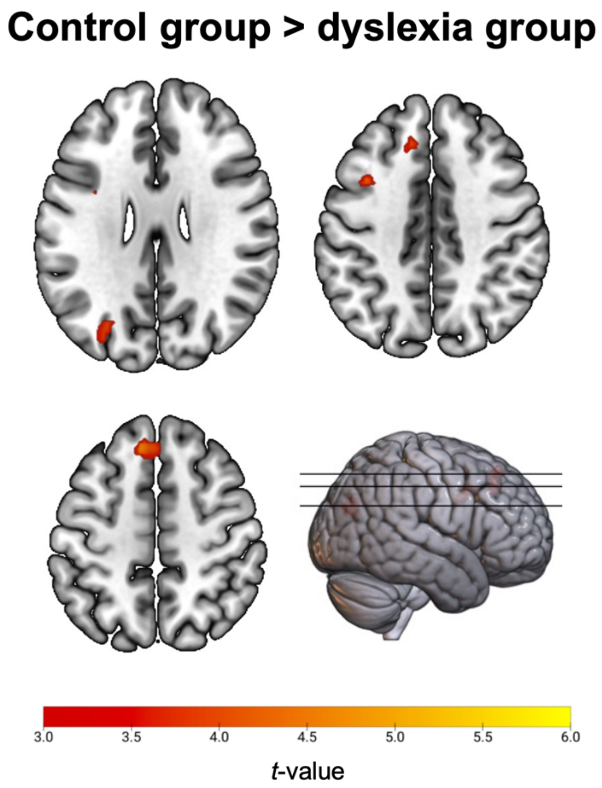

Supplementary Figure 4. Significant between-group fMRI activations based on t-statistic maps during the rhyming task for controls (n = 35) > dyslexia (n = 35) with voxel-level threshold: $p < 0.001$ uncorrected, cluster-level threshold: $p < 0.05$ FWE-corrected. Colour scale bar indicates the magnitude of t-values.

C. Group-level and between-group fMRI activations during the fluency task

Contrast	Anatomical definition	MNI coordinates (peak voxel)			t-value	Cluster size
		X	Y	Z		
**Wordgen Control**	**Right lobule VI of cerebellar hemisphere**	**26**	**-62**	**-26**	**16.06**	**18154**
	Right crus I of cerebellar hemisphere	44	-60	-30	15.66	
	Left supplementary motor area	-6	8	60	15.5	
	**Left inferior parietal gyrus**	**-48**	**-40**	**47**	**9.59**	**1082**
	Left superior parietal gyrus	-28	-62	47	8.64	
	Left middle occipital gyrus	-24	-72	30	7.71	
	**Left lobule IX of cerebellar hemisphere**	**-6**	**-37**	**-43**	**7.59**	**184**
	Left lobule X of cerebellar hemisphere	-1	-30	-48	6.82	
	Left lobule IX of cerebellar hemisphere	-4	-44	-56	5.57	
	**Right middle frontal gyrus**	**39**	**46**	**20**	**5.66**	**147**
	Right middle frontal gyrus	32	43	12	5.28	
	Right middle frontal gyrus	32	30	22	4.34	
	**Right angular gyrus**	**29**	**-50**	**32**	**5.48**	**69**
	Right precuneus	22	-50	44	4.65	
	Right angular gyrus	32	-60	40	3.91	
**Wordgen Dyslexia**						
	**Right lobule VIII of cerebellar hemisphere**	**29**	**-67**	**-56**	**15.09**	**1377**
	Right crus I of cerebellar hemisphere	44	-62	-28	13.59	
	Right lobule VI of cerebellar hemisphere	26	-64	-23	12.87	
	**Left insula**	**-31**	**26**	**2**	**14.67**	**5913**
	Right insula	32	23	2	12.71	
	Left inferior frontal gyrus, pars orbitalis	-41	26	-6	11.04	
	**Left supplementary motor area**	**-4**	**16**	**44**	**12.62**	**2007**
	Left superior frontal gyrus, medial	-4	23	37	12.11	
	Left supplementary motor area	-1	8	60	12.09	
	**Left crus I of cerebellar hemisphere**	**-48**	**-60**	**-30**	**6.55**	**189**
	Left lobule VI of cerebellar hemisphere	-34	-54	-30	6.47	
	**Left middle occipital gyrus**	**-28**	**-67**	**40**	**6.4**	**222**
	Left inferior parietal gyrus	-28	-60	44	5.21	
	Left angular gyrus	-28	-54	30	4.86	
	**Left middle temporal gyrus**	**-61**	**-32**	**4**	**6.37**	**104**
	**Left inferior parietal gyrus**	**-44**	**-40**	**42**	**5.85**	**96**
	Left inferior parietal gyrus	-51	-34	44	4.77	
	**Left putamen**	**-34**	**-20**	**-8**	**5.72**	**106**
	Left hippocampus	-28	-34	7	5.38	
	Left hippocampus	-34	-32	0	4.44	
	**Right calcarine fissure and surrounding cortex**	**32**	**-52**	**7**	**5.45**	**68**
	Right hippocampus	34	-44	4	4.3	
	Right hippocampus	34	-34	0	4.02	
	**Left middle frontal gyrus**	**-31**	**46**	**20**	**5.15**	**81**
	Left middle frontal gyrus	-36	53	20	4.49	
**CON>DYS**	**Right lobule III of cerebellar hemisphere**	**14**	**-37**	**-23**	**5.44**	**93**
	Right lobule IX of cerebellar hemisphere	14	-50	-30	4.33	
	Right lobule IV, V of cerebellar hemisphere	22	-37	-28	4.25	
	**Right precentral gyrus**	**22**	**-4**	**47**	**4.74**	**65**
	Right precentral gyrus	32	-10	54	3.76	
	**Left middle frontal gyrus**	**-31**	**3**	**34**	**4.62**	**129**
	Left precentral gyrus	-46	0	20	4.26	
	Left precentral gyrus	-46	6	34	4.22	
	**Right angular gyrus**	**24**	**-50**	**42**	**4.38**	**93**
	Right angular gyrus	29	-50	34	4.21	
	Right inferior parietal gyrus	36	-42	47	4.12	
**DYS>CON**		-	-	-	-	-

Supplementary Table 3. Whole-brain analysis results based on t-statistic maps for the contrast fluency > control in the control group (CON, n = 35) and the dyslexia group (DYS, n = 35), as well as between-group comparisons (CON > DYS and DYS > CON). Significant activations were identified at a voxel-level threshold of $p < .001$ (uncorrected) with cluster-level FWE correction at $p < .05$.

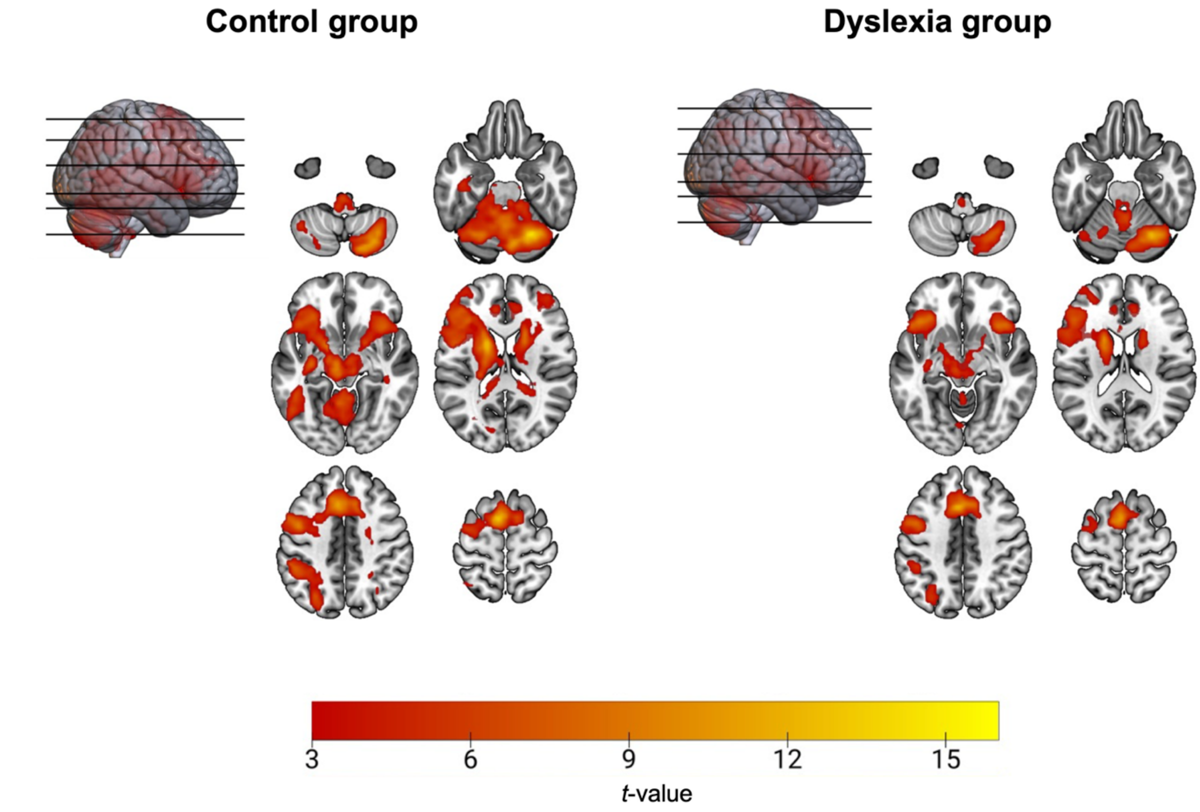

Supplementary Figure 5. Significant fMRI activations based on t-statistic maps during the fluency task shown separately for the control group (n = 35) and the dyslexia group (n = 35). Statistical maps were thresholded at a voxel-level of $p < .001$ (uncorrected) and a cluster-level of $p < .05$ (FWE-corrected). Colour scale bar indicates the magnitude of t-values.

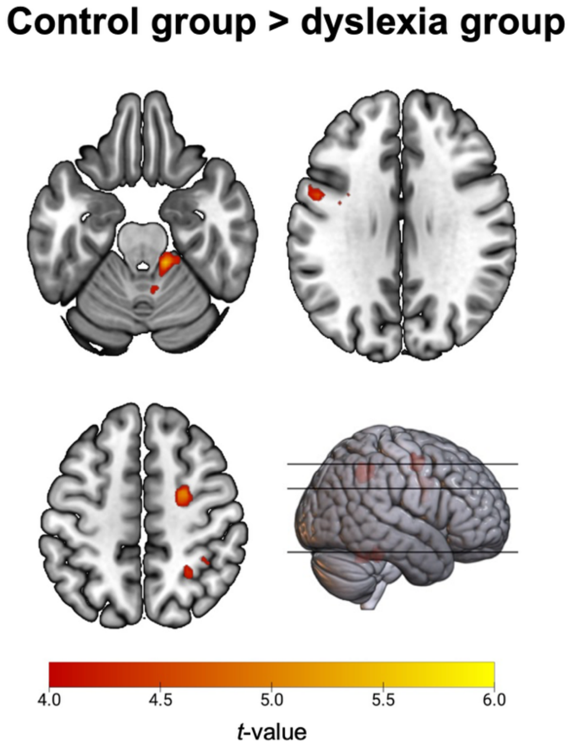

Supplementary Figure 6. Significant between-group fMRI activations based on t-statistic maps during the fluency task for controls (n = 35) > dyslexia (n = 35) with voxel-level threshold: $p < 0.001$ uncorrected, cluster-level threshold: $p < 0.05$ FWE-corrected. Colour scale bar indicates the magnitude of t-values.

D. Mean laterality indices (LIs), standard deviations, and one-sample tests against zero for each ROI

Task	ROI	Group	Mean LI	SD	*t*-value	*P*-value
**Reading task**	Composite ROI	CON	-0.61	0.13	-27.67	**< 0.001**
		DYS	-0.52	0.25	-12.18	**< 0.001**
	Fusiform gyrus	CON	-0.54	0.13	-24.64	**< 0.001**
		DYS	-0.30	0.35	-5.13	**< 0.001**
	Inf frontal gyrus	CON	-0.57	0.16	-20.75	**< 0.001**
		DYS	-0.47	0.26	-10.50	**< 0.001**
	Inf occipital gyrus	CON	-0.48	0.22	-12.64	**< 0.001**
		DYS	-0.34	0.37	-5.45	**< 0.001**
	Inf parietal gyrus	CON	-0.42	0.33	-7.56	**< 0.001**
		DYS	-0.24	0.43	-3.33	**0.002**
	Mid temporal gyrus	CON	-0.52	0.25	-12.29	**< 0.001**
		DYS	-0.45	0.32	-8.18	**< 0.001**
	Precentral gyrus	CON	-0.57	0.16	-20.79	**< 0.001**
		DYS	-0.55	0.22	-14.64	**< 0.001**
**Rhyming task**	Composite ROI	CON	-0.67	0.08	-48.45	**< 0.001**
		DYS	-0.59	0.28	-12.67	**< 0.001**
	Cerebellum	CON	0.44	0.25	10.54	**< 0.001**
		DYS	0.38	0.30	7.43	**< 0.001**
	Inf frontal gyrus	CON	-0.66	0.08	-49.60	**< 0.001**
		DYS	-0.59	0.26	-13.65	**< 0.001**
	Mid temporal gyrus	CON	-0.45	0.22	-11.84	**< 0.001**
		DYS	-0.36	0.39	-5.50	**< 0.001**
	Precentral gyrus	CON	-0.66	0.06	-62.56	**< 0.001**
		DYS	-0.58	0.27	-12.76	**< 0.001**
	Sup motor area	CON	-0.49	0.20	-14.67	**< 0.001**
		DYS	-0.37	0.32	-6.91	**< 0.001**
**Fluency task**	Composite ROI	CON	-0.68	0.07	-57.59	**< 0.001**
		DYS	-0.61	0.30	-12.05	**< 0.001**
	Cerebellum	CON	0.57	0.14	23.38	**< 0.001**
		DYS	0.52	0.25	12.19	**< 0.001**
	Inf frontal gyrus	CON	-0.66	0.08	-49.81	**< 0.001**
		DYS	-0.60	0.31	-11.41	**< 0.001**
	Precentral gyrus	CON	-0.66	0.12	-33.79	**< 0.001**
		DYS	-0.60	0.24	-14.90	**< 0.001**
	Sup motor area	CON	-0.55	0.11	-30.17	**< 0.001**
		DYS	-0.47	0.27	-10.14	**< 0.001**
	Thalamus	CON	-0.47	0.19	-14.70	**< 0.001**
		DYS	-0.38	0.31	-7.24	**< 0.001**

Supplementary Table 4. Mean LIs, standard deviations, and one-sample tests against zero using original values. These tests confirm that both the dyslexia group and the control group are lateralized for each ROI.

E. Independent-samples *t*-tests comparing laterality indices (LIs) between the dyslexia and control groups using original LI values

Task	ROI	*t*-value	df	*P*-value	Effect size (d)
**Reading task**	Composite ROI	-1.75	50.64	**0.043**	-0.42
	Fusiform gyrus	-3.81	43.29	**0.001**	-0.91
	Inf frontal gyrus	-1.93	56.51	**0.030**	-0.46
	Inf occipital gyrus	-1.87	55.92	**0.033**	-0.45
	Inf parietal gyrus	-1.97	63.85	**0.027**	-0.47
	Mid temporal gyrus	-1.08	64.10	0.143	-0.26
	Precentral gyrus	-0.55	62.53	0.291	-0.13
**Rhyming task**	Composite ROI	-1.56	39.87	0.064	-0.37
	Cerebellum	0.94	65.49	0.176	0.22
	Inf frontal gyrus	-1.53	40.37	0.067	-0.37
	Mid temporal gyrus	-1.11	54.13	0.135	-0.27
	Precentral gyrus	-1.65	37.62	0.054	-0.39
	Sup motor area	-1.82	56.59	**0.037**	-0.43
**Fluency task**	Composite ROI	-1.31	37.67	0.100	-0.31
	Cerebellum	0.96	53.90	0.170	0.23
	Inf frontal gyrus	-1.26	38.41	1.107	-0.30
	Precentral gyrus	-1.33	49.13	0.096	-0.32
	Sup motor area	-1.64	44.32	0.054	-0.39
	Thalamus	-1.47	54.72	0.074	-0.36

Supplementary table 5. Statistics from t-tests comparing the LI values for the dyslexia and control group using original LI values (see Supplementary table 1). Note that the dyslexia group appear less lateralized for several regions, when not controlling for direction of asymmetry, most notably in the reading task.
